# Genome-Wide Identification and Evaluation of New Reference Genes for Gene Expression Analysis Under Temperature and Salinity Stresses in *Ciona savignyi*

**DOI:** 10.3389/fgene.2019.00071

**Published:** 2019-02-12

**Authors:** Xuena Huang, Shiguo Li, Aibin Zhan

**Affiliations:** ^1^Key Laboratory of Environmental Biotechnology, Research Center for Eco-Environmental Sciences, Chinese Academy of Sciences, Beijing, China; ^2^University of Chinese Academy of Sciences, Chinese Academy of Sciences, Beijing, China

**Keywords:** biological invasion, *Ciona savignyi*, reference gene, real time PCR, environmental stress, gene expression, RNA-Seq

## Abstract

Rapid adaptation/accommodation to changing environments largely contributes to maximal survival of invaders during biological invasions, usually leading to success in crossing multiple barriers and finally in varied environments in recipient habitats. Gene expression is one of the most important and rapid ways during responses to environmental stresses. Selection of proper reference genes is the crucial prerequisite for gene expression analysis using the common approach, real-time quantitative PCR (RT-qPCR). Here we identified eight candidate novel reference genes from the RNA-Seq data in an invasive model ascidian *Ciona savignyi* under temperature and salinity stresses. Subsequently, the expression stability of these eight novel reference genes, as well as other six traditionally used reference genes, was evaluated using RT-qPCR and comprehensive tool RefFinder. Under the temperature stress, two traditional reference genes, ribosomal proteins S15 and L17 (*RPS15, RPL17*), and one novel gene Ras homolog A (*RhoA*), were recommended as the top three stable genes, which can be used to normalize target genes with a high and moderate expression level, respectively. Under the salinity stress, transmembrane 9 superfamily member (*TMN*), MOB kinase activator 1A-like gene (*MOB*) and ubiquitin-conjugating enzyme (*UBQ2*) were suggested as the top three stable genes. On the other hand, several commonly used reference genes such as α-tubulin (*TubA*), β-tubulin (*TubB*) and glyceraldehyde-3-phosphate dehydrogenase (*GAPDH*) showed unstable expressions, thus these genes should not be used as internal controls for gene expression analysis. We also tested the expression level of an important stress response gene, large proline-rich protein bag6-like gene (*BAG*) using different reference genes. As expected, we observed different results and conclusions when using different normalization methods, thus suggesting the importance of selection of proper reference genes and associated normalization methods. Our results provide a valuable reference gene resource for the normalization of gene expression in the study of environmental adaptation/accommodation during biological invasions using *C. savignyi* as a model.

## Introduction

Biological invasion has been recognized as one of the major ecological and environmental problems in many ecosystems globally, causing significantly negative ecological and economic impacts (Simberloff et al., 2013). The invasion success and subsequent spreading magnitude of a species highly depend on many influential factors, such as species characteristics, environmental features of donor and recipient habitats, and introduction/spread vectors as well (Kolar and Lodge, 2001; Zhan et al., 2015). Among these factors, the ability of rapid adaptation/accommodation to changing or novel environments during biological invasions largely contributes to the successful crossing of multiple barriers during the process of biological invasions and the final success in varied environments of recipient habitats (Kolar and Lodge, 2001; Zhan et al., 2015). Along with many other regulation mechanisms at the molecular level such as metabolism alteration, protein homeostasis, regulation of enzymatic activities and epigenetic modification, gene expression is a rapid and functional process responsible for environmental adaptation/accommodation (Nadal et al., 2011). Gene expression regulation can provide an immediate and reversible response to various environmental stresses, and such a functional regulation is essential for invasive species to survive rapidly changing environments and/or novel environments during habitat transitions.

The commonly used technique for gene expression analysis is real-time quantitative PCR (RT-qPCR), mainly owing to its advantages of sensitivity, specificity and rapid execution (Pombo et al., 2017). To ensure the accuracy of relative expression changes among samples, reference genes are required for RT-qPCR to avoid potential artifacts caused by sample preparation and detection (Li et al., 2015). The mRNA expression of an ideal reference gene should have minimal or no variation in different tissues, developmental stages, and diverse environmental conditions. However, many studies found that the traditionally used reference genes were unstably expressed in different experimental conditions, and conclusions made based on target gene expression changes normalized with unstable reference genes might be biased or even totally wrong. For example, the commonly used reference gene, glyceraldehyde-3-phosphate dehydrogenase (*GAPDH*), was not recommended in gene expression analysis in different developmental stages in the insect *Aedes aegypti* (Dzaki et al., 2017), and both *GAPDH* and actin (*ACT*) genes were identified as the least stable reference genes during developmental stages and abiotic stresses in the grass *Setaria viridis* (Martins et al., 2016). In addition, *GAPDH* and α-tubulin (*TubA*) were unstably expressed genes in the fish *Odontesthes humensis* after different environmental stress challenges (Silveira et al., 2018). Therefore, prudent selection and validation of reliable reference genes for specific experimental conditions is the crucial prerequisite for accurate measurement of target gene expression.

Evaluating the stability of pre-selected candidate reference genes using RT-qPCR is the most frequently used method for reference genes selection (Amil-Ruiz et al., 2013; Martins et al., 2016; Chong et al., 2017; Dzaki et al., 2017). However, this method is overwhelmingly dependent on the availability of pre-selected genes, and such a method may neglect the genes that would have been the most stable reference genes for a given experimental condition (Zhao et al., 2017). Additionally, the traditional method relies on available candidate genes derived from related studies, thus cannot identify any novel stably expressed genes. With the rapid development of next-generation sequencing technologies, gene expression quantification by the strategy of whole transcriptome sequencing (i.e., RNA-Seq) is a powerful approach for the analysis of gene expression (Hoang et al., 2017). RNA-Seq allows accurate measurement of gene expression levels with a large dynamic range of expression, but most of the RNA-Seq studies mainly focused on the differentially expressed genes (DEGs) whose functions are assumed to be involved in an organism under specific experimental conditions. In addition to DEGs, constitutively expressed genes can be used to screen stable expression genes suitable for RT-qPCR. In recent years, RNA-Seq data has been used for the selection and validation of more new stable reference genes in many studies, for example in pregnant woman (Chim et al., 2017), human cancer tissue (MacRae et al., 2013), different *Streptomyces coelicolor* strains (Li et al., 2015), and different developmental stages and drought stress conditions in Goji (Gong et al., 2016). These studies successfully identified new reference genes which are more stable than the traditionally used ones, indicating that selection of stably expressed reference gene from RNA-Seq data is a powerful and reliable method.

As a marine invasive species widely distributed in north Asia, Pacific coast of North America and New Zealand (Lambert and Lambert, 1998; Zvyagintsev et al., 2007), *Ciona savignyi* has become an excellent model system for studying invasion success (Zhan et al., 2015). According to our previous study, *C. savignyi* could rapidly respond to environmental changes such as those crucial for many marine organisms including temperature and salinity at the DNA methylation level (Huang et al., 2017). Due to the regulation relationship between DNA methylation and gene expression, we hypothesized that *C. savignyi* could also rapidly respond to changing environments at the gene expression level (Huang et al., 2017). Indeed, we have recently found that an important category of stress response genes, heat shock proteins (*Hsp*), could respond to environmental challenges by up- or down-regulation of their mRNA expressions by RNA-Seq analysis (Huang et al., 2018).

In order to obtain accurate results for systematic gene expression analysis, here we used a large set of RNA-Seq data to choose stable reference genes for two environmental factors (i.e., temperature and salinity) that *C.* *savignyi* frequently encounters during invasion processes among different habitats. We aim to select a valuable set of reliable reference genes for studying the role of gene expression in rapid environmental adaptation/accommodation during biological invasions using *C. savignyi* as a model. Subsequently, the stability of newly selected and other commonly used reference genes was further validated by RT-qPCR using the online tool RefFinder, and finally all selected reference genes were re-assessed in the expression analysis of one target gene, large proline-rich protein bag6-like gene (*BAG*) which participates in maintenance of protein homeostasis under stressful conditions, thus might play an important role in rapid adaption to changing environments for invasive ascidians (Hessa et al., 2011). The validated reference genes here are expected to serve as reliable resources to further investigate molecular mechanisms of evolutionary adaptation and/or accommodation at the population level using the fast and accurate RT-qPCR method.

## Materials and Methods

### Animal Collection and Environmental Treatments

Adult *Ciona savignyi* individuals were collected from scallop cages along the coast of Dalian, Liaoning Province, China. After 1 week of acclimation in the laboratory, healthy ascidians were randomly assigned to one of five experimental treatments: control (C), high temperature (HT), low temperature (LT), high salinity (HS) and low salinity (LS). The temperature and salinity parameters of control group were set as 15°C and 30‰, respectively, according to the field conditions of the sampling site. HT and LT were a rise or decrease by 10 degrees in temperature-controlled tanks at the rate of 6°C per hour from 15°C with unchanged salinity (i.e., 25°C/30‰ and 5°C/30‰). Accordingly, HS and LS were set as 15°C/40‰ and 15°C/20‰, respectively, and individuals were abruptly subjected to the pre-set target salinity. According to our previously reported results (Huang et al., 2017), we chose three key duration time points after five treatments, 1, 24, and 48 h, to analyze the transcriptional response mechanisms. Pharynx tissue from three biological replicates of each group (five groups × three time points × three biological replicates = 45 samples) were dissected and immediately preserved in liquid nitrogen, and then stored at -80°C until RNA isolation and gene expression analysis.

### Total RNA Isolation and cDNA Synthesis

Total RNA was extracted by the TRIZOL reagent (Ambion, United States) according to the manufacturer’s instruction. The integrity of the extracted total RNA was preliminarily tested by visual inspection of the 28S and 18S ribosomal bands using 1.5% agarose gel electrophoresis. For the following RNA sequencing analysis, RNA concentration and integrity were further examined using Agilent 2100 BioAnalyzer (Agilent, United States) and only samples with RNA Integrity Number (RIN) > 7.5 were used for cDNA library construction. For RT-qPCR analysis, RNA purity and quantity were assessed using a Nanodrop 2000 spectrophotometer (Nanodrop Technologies, United States), and subsequently qualified RNA samples were treated with RNase-free DNase I (Promega, United States) to eliminate genomic DNA contamination. The first-strand cDNA was synthesized from 1.5 μg of treated total RNA using M-MLV reverse transcriptase (Takara, Japan) with Oligo-dT primer according to standard procedures. The cDNA was stored at -20°C until use.

### RNA Sequencing and Gene Expression Analysis

A total of 45 cDNA libraries (5 treatments × 3 duration time points × 3 biological replicates) were constructed using NEBNext UltraTM RNA Library Prep Kit (NEB^®^, United States) and sequenced on the HiSeq4000 platform (Illumina, United States) with the pair end of 150 bp strategy. Raw reads were checked by FastQC, and then the adapter and the low-quality sequences were filtered out and trimmed. The obtained clean reads were mapped to the *C. savignyi* reference genome using the HISAT2 with the following parameters: -p (threads) 8 -N (max mismatches in seed alignment) 1 -L (length of seed substrings) 20 -i (interval between seed substrings w/r/t read) S, 1, 0.5 -D (give up extending after 25 failed extends in a row) 25 -R (for reads w/ repetitive, try 5 sets of seeds) 5 –mp (set the maximum and minimum mismatch penalties) 1, 0 -sp (set the maximum and minimum penalties for soft-clipping per base) 3, 0 -x hisat2_index. The mapped reads were then assembled into transcripts and calculated as gene expression level using StringTie (Pertea et al., 2016). The value of fragments per kilobase per million reads (FPKM) was used to represent the expression level of each gene.

### Novel Candidate Reference Genes Screening Based on RNA-Seq Data

We firstly removed the genes that were not detected in any of 45 samples based on FPKM values, and finally obtained 8320 constitutively expressed genes whose FPKM values are greater than 1 in all 45 samples. The coefficient of variation (CV, the ratio of the standard deviation (SD) to the arithmetic means) for each gene among 45 samples was used as the criteria to evaluate the expression stability of genes. The CV and mean of FPKM value of each gene was visualized by scatterplot. The genes with CV of FPKM lower than 20%, which means a gene with least variance among samples, were chosen for functional analysis through GO and KEGG enrichment of representation of gene ontology terms and pathways. The first 11 genes with the CV value lower than 16%, including ubiquitin carboxyl terminal hydrolase gene: *UBQ1*; zinc finger protein: *ZNF*; conserved oligomeric Golgi complex subunit 1-like: *COG*; ras-related protein ORAB: *RAB*; numb protein: Numb; transmembrane 9 superfamily member: *TMN*; cell division cycle 42: *CDC42*; ADP-ribosylation factor-like protein 1: *ARL*; transmembrane emp24 domain-containing protein 1-like: *TMED*; RhoA protein: *RhoA*; MOB kinase activator 1A-like: *MOB*, and another six commonly used reference genes, including ubiquitin-conjugating enzyme E2 K-like: *UBQ2*; ribosomal protein S15: *RPS15*; ribosomal protein L17: *RPL17*; α-tubulin: *TubA*; β-tubulin: *TubB*; *GAPDH* (Huang et al., 2016), were selected to show the variability of FPKM based on RNA-Seq results by boxplot and then further assessed by RT-qPCR.

### The Relationship Between Gene Expression Stability and Gene Structure

The gene structure information including GC content, transcript length, exon count, coding sequence length, transcript count and gene length of 8320 genes was extracted using BioMart tool at the Ensemble website. Using the method by Carmel and Koonin (2009), genes were divided into 42 groups according to the order of average CV (or FPKM) values from high to low. The number of genes in each group was kept as closely as possible. The gene structure difference between group pairs was statistically test using Mann–Whitney *U* test.

### RT-qPCR and Data Analysis

The gene expression stability of new candidate reference genes selected based on RNA-Seq data and other commonly used reference genes were further analyzed by RT-qPCR. Firstly, the primers of 11 new candidate reference genes for RT-qPCR were designed using Primer Premier 5.0^1^. The amplification specificity of all primer pairs was tested by 1.5% agarose gel electrophoresis and primers validity was verified by Sanger sequencing. RT-qPCR of gene expression was performed using Roche LightCycler^®^96 detection system (Roche Applied Science, Germany) with FastStart Essential DNA Green Master (Roche Applied Science, Germany). A total volume of 10 μL of PCR reaction mix contained 5.0 μL of SYBR Green Master Mix, 0.5 μL (10 pmol) of forward and reverse primers and 1 μL cDNA (10x dilution). Each amplification was performed in triplicate and the PCR thermocycling condition was 95°C for 10 min, followed by 40 cycles of denaturation at 95°C for 10 s, annealing at 60°C for 10 s and extension at 72°C for 15 s. Fluorescent signals were collected at each extension stage. Dissociation analysis of amplification products was performed at the end of each PCR reaction to confirm that only the specific PCR product was amplified and detected. The amplification efficiency (E) of each candidate reference gene was calculated by the slope of its standard curve which was generated using 10-fold serial dilutions of the same cDNA samples. The correlation coefficient (*R*^2^) was determined by the linear-correlation of regression analysis to validate the linear relationship between the quantification cycle values (Cq) and the template concentration.

### Evaluation of Gene Expression Stability

The Cq values of all candidate reference genes, which were generated by the raw fluorescence data produced during the qRT-PCRs, were used to represent the gene expression level. The expression stability of candidate reference genes was evaluated by the online tool RefFinder (Xie et al., 2011), which integrated four commonly used programs, geNorm version 3.5 (Vandesompele et al., 2002), NormFinder version 0.953 (Andersen et al., 2004), comparative delta Ct method (Silver et al., 2006) and BestKeeper (Pfaffl et al., 2004).

### Target Gene Expression Analysis

After evaluating the expression stability of all candidate reference genes as described above, we selected an important stress response gene, large proline-rich protein bag6-like gene (*BAG*: ENSCSAVG00000006988), from DEG list identified by RNA-Seq to validate the selected reference genes. According to the equation ΔΔCq = (Cq_target_ – Cq_reference_)_treatment_ – Average (Cq_target_ – Cq_reference_)_control_, the expression level of target gene in non-treatment sample (control) was set to a value of 1, the expression results of post-treatment samples represented fold change value relative to the corresponding control sample, and the positive or negative nature of the ΔΔCq values indicated up- or down-regulation of target gene differential expression. We then compared the expression of BAG gene normalized by selected reference genes with its expression results obtained from RNA-Seq.

## Results

### Shortlist of Candidate Genes

**FIGURE 1:**
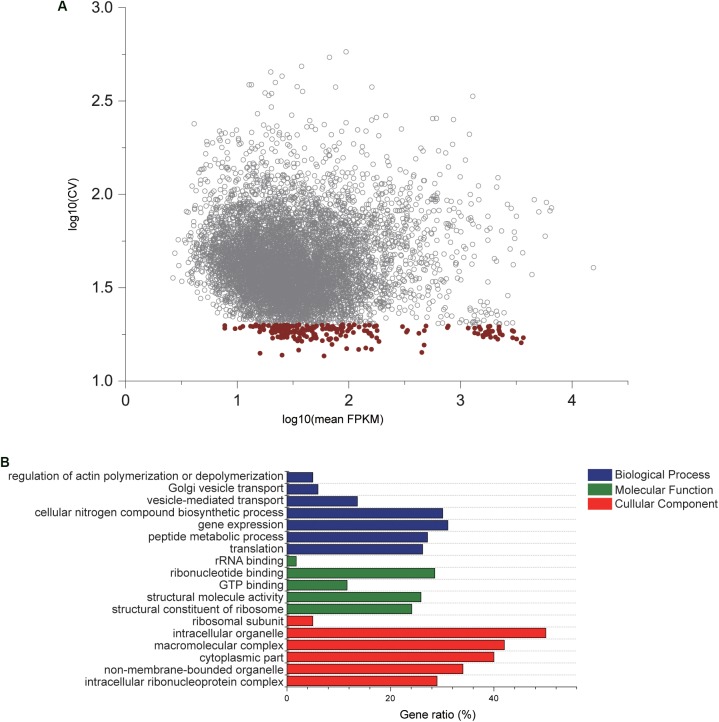
Gene expression characteristics and GO enrichment analysis of candidate reference genes. **(A)** Scatterplot of the coefficient of variation × 100% (CV%) against mean expression values (FPKM) after log_10_ transformed based on RNA-Seq data. Each circle represents a gene, and red circles are 196 genes with CV lower that 20%. **(B)** Significantly enriched GO terms by GO enrichment analysis conducted with 196 genes. Gene ratio is defined as the percentage of genes in the GO terms that overlaps with the gene shortlist.

After genome-wide RNA sequencing of 45 *C. savignyi* samples under temperature or salinity stresses, we successfully assembled all predicted genes (12172) in its reference genome. The number of raw or clean reads and mapping rate of each sample were listed in Supplementary Table S1. The FPKM values of each gene among different samples were used to evaluate the stable expression characteristics. A reference gene should express at the detectable level, thus a total of 8320 (68.36%) genes were remained for downstream analyses after removing those with undetectable expression in any of the 45 samples. The average FPKM values ranged from 2.65 to 15493.79, and the coefficient of variation (CV) ranged from 13.63% to 581.24% (Figure 1A). After applying the criteria for CV of FPKM < 20%, 196 genes were selected as relatively stable ones for further analyses (Figure 1A). GO enrichment analysis on this set of 196 genes revealed that these stably expressed genes were significantly enriched to or mainly associated with basic cellular components such as ribosomal subunit or cytoplasmic part, molecular functions like GTP binding, and biological processes such as gene expression and translation (adjusted *p*-value < 0.05) (Figure 1B). KEGG pathway enrichment analysis revealed that these genes were involved in critical cellular basic functions including ribosome and endocytosis significantly (adjusted *p*-value < 0.05). This shortlist provides candidate reference genes for studying response mechanisms associated with temperature and salinity stresses using *C. savignyi* as a model.

### Relationship Between Expression Level and Gene Structure

**FIGURE 2:**
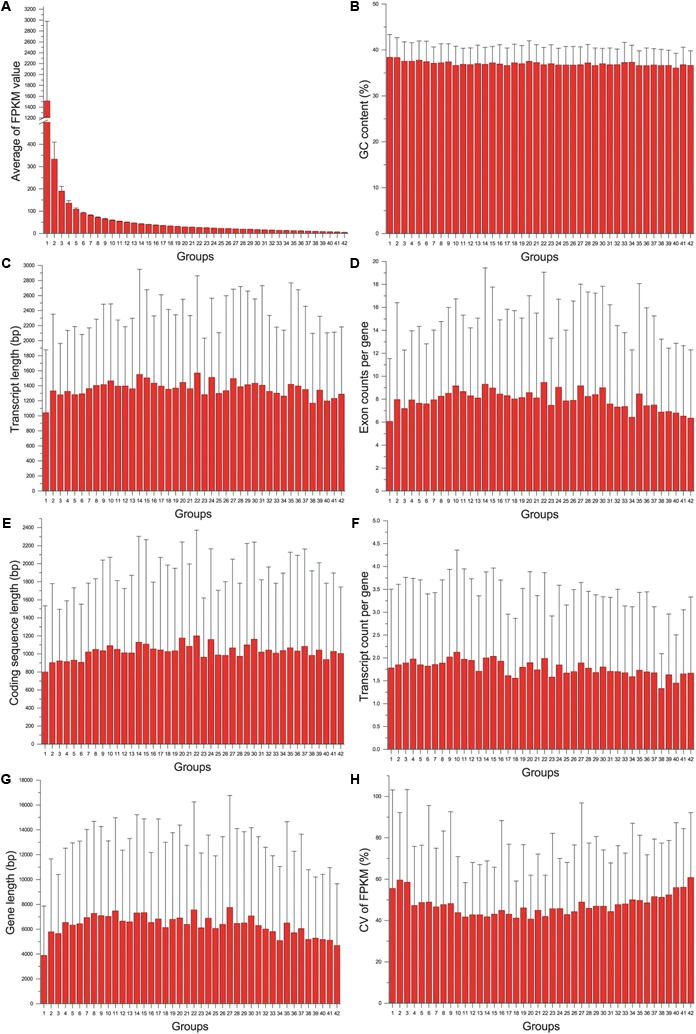
The relationship between gene structure and expression level: the distribution of average of FPKM value **(A)**, GC content **(B)**, transcript length **(C)**, Exon count per gene **(D)**, coding sequence length **(E)**, transcript count per gene **(F)**, gene length **(G)**, and CV of FPKM **(H)** across different groups.

The gene expression level (average FPKM) of 8320 genes was not significantly correlated with their variance (CV) (Pearson correlation coefficient = 0.021, *p* = 0.055). Although both expression level and variance were significantly correlated with several variables of gene structure, the correlation coefficient was too low (lower than 0.12). When genes were grouped according to their CV, no particular trends were found by all the gene structure parameters. While genes were grouped according to their expression levels, we found that the genes in the first group (with highest gene expression level, Figure 2A) had the shortest transcript length and coding sequence length (Figure 2C,E), and had shorter gene length and less exon count (Figure 2G,D). However, the similar relationship was not detected between gene expression level and GC content or transcript count (Figure 2B,F). In addition, we also found that genes with higher or lower expression levels tended to be more unstable than genes with a medium expression level (Figure 2H), indicating that reference genes should be selected from moderately expressed genes.

### Selection of Novel Candidate Reference Genes

**FIGURE 3:**
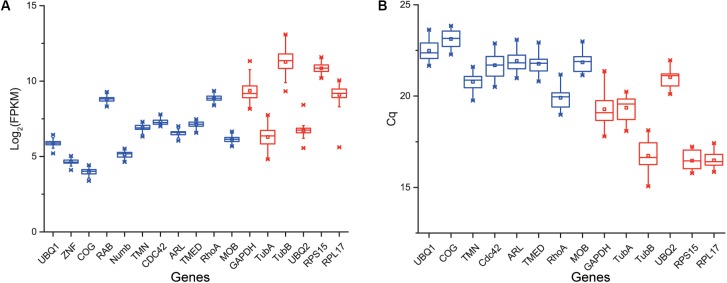
Boxplot showing the distribution of expression level (FPKM) of 11 novel candidate reference genes (blue) and six commonly used reference genes (red) based on RNA-Seq data (*n* = 45) **(A)**, and the distribution of expression level (Cq) of eight novel candidate reference genes (blue) and six commonly used reference genes (red) based on RT-qPCR **(B)**. Whisker caps represent maximum and minimum values, the upper and lower lines of the box represent the 25/75 percentiles, the line across the box represents the median, and the asterisk indicates outliers.

According to the rank of gene expression stability based on CV of FPKMs, we selected 11 genes (Table 1) with the lowest CV (i.e., the most stable genes suggested by FPKM). Interestingly, most of these genes have not been used previously as reference genes in RT-qPCR analysis except for the *UBQ1* and *RhoA*. For this set of genes, CV of FPKM ranged from 13.63% to 15.73%. We also selected six commonly used reference genes identified previously in *C. savignyi’s* transcriptional regulation study under temperature and salinity stresses (Huang et al., 2016), including *RPS15, RPL17, UBQ2, GAPDH, TubA* and *TubB*. The newly identified reference genes were more stably expressed than those previously reported genes based on CV of FPKM, but the latter generally showed a higher expression level (Table 1 and Figure 3A).

**Table 1:** Detailed information of selected candidate reference genes and commonly used reference genes based on RNA-Seq data.

Gene ID	Gene name	Gene abbreviation	Mean FPKM	CV%
ENSCSAVG00000001569	Ubiquitin carboxyl-terminal hydrolase	*UBQ1*	56.69	13.63
ENSCSAVG00000001885	Zinc finger protein	*ZNF*	25.14	13.78
ENSCSAVG00000006667	Conserved oligomeric Golgi complex subunit 1-like	*COG*	15.94	14.11
ENSCSAVG00000009147	ras-related protein ORAB	*RAB*	448.25	14.24
ENSCSAVG00000011796	numb protein	*Numb*	35.41	14.67
ENSCSAVG00000008637	Transmembrane 9 superfamily member	*TMN*	122.14	14.76
ENSCSAVG00000011760	Cell division cycle 42	*CDC42*	159.01	14.81
ENSCSAVG00000007620	ADP-ribosylation factor-like protein 1	*ARL*	95.46	14.92
ENSCSAVG00000007485	Transmembrane emp24 domain-containing protein 1-like	*TMED*	141.15	15.06
ENSCSAVG00000006924	RhoA protein	*RhoA*	470.17	15.60
ENSCSAVG00000011530	MOB kinase activator 1A-like	*MOB*	70.50	15.73
ENSCSAVG00000008262	Ribosomal protein S15	*RPS15*	436.12	23.04
ENSCSAVG00000010299	Ribosomal protein L17	*RPL17*	204.06	34.21
ENSCSAVG00000009208	Ubiquitin-conjugating enzyme E2 K-like	*UBQ2*	40.25	36.56
ENSCSAVG00000009748	α-tubulin	*TubA*	40.96	46.96
ENSCSAVG00000004683	β-tubulin	*TubB*	1419.67	51.02
ENSCSAVG00000007442	Glyceraldehyde-3-phosphate dehydrogenase	*GAPDH*	503.90	66.21

### Gene Expression Stability Analysis by RT-qPCR

We validated and compared the expression stability of novel and commonly used reference genes by RT-qPCR. Firstly, we designed gene-specific primers for 11 novel candidate reference genes, but three of them (*ZNF, RAB, Numb*) did not pass tests because of non-specific amplification or low amplification efficiency. The PCR amplification efficiency of the remaining eight primer pairs ranged from 1.94 to 2.07 (Table 2). The primers of six commonly used reference genes were employed from a previous report (Huang et al., 2016). The raw quantification cycle values (Cq) of 14 genes among all samples were analyzed for an overview of expression level and variation degree (Table 2 and Figure 3B). We found that the expression level (mean Cq value) of six commonly used reference genes was significantly higher than that of eight novel reference genes (Mann–Whitney *U* test, *p* < 0.05), and *RPS15* showed the highest expression level with an average Cq of 16.46. However, the ranks of CV values were mixed between novel and commonly used reference genes. When the expression stability of reference genes was evaluated by the standard deviation (SD) of Cq, *RPL17* (0.39) and *GAPDH* (0.84) showed the most and the least stability, respectively (Table 2). Additionally, we found that the expression level obtained from RT-qPCR (mean Cq) was significantly correlated with the results of RNA-Seq represented by mean FPKM (correlation coefficient = -0.645, *p* < 0.05), indicating that these two gene expression quantitative methods are basically consistent with each other.

**Table 2:** Primer sequences and PCR amplification parameters of reference genes and target genes derived from RT-qPCR analysis.

			Product	Amplification	Mean	Standard		Rank
Genes	Forward primer	Reverse primer	length (bp)	efficiency (%)	Cq	Deviation (SD)	CV%	by CV
*UBQ1*	GTGCTCATTAAAGTCCGTACCGG	TACCTGTCCACCTACAACCACAG	126	2.07	22.48	0.52	2.31	4
*COG*	CAGCACGAAGAAAAGGAACGCG	CGGTATAAGACGACGACATTGAC	104	1.99	23.12	0.47	2.03	1
*TMN*	GACAGATCCCAGAACAAGTGC	TGGTGGGACCATATTGAGTTCAG	124	2.06	20.78	0.45	2.16	2
*CDC42*	GTGCTCTGCCCTTACTCAACG	CAACTGAGCAACCGCCCTTTT	104	1.98	21.69	0.70	3.23	12
*ARL*	CTACAAGTTGGTGAGGTGGTG	ACTGGTCTGTCCGCCTAAATC	111	2.01	21.92	0.59	2.69	8
*TMED*	GCCAGCAGGCAAGAAAGAATGT	CACGACCAGATGGAGCAGAAAT	128	1.97	21.77	0.57	2.62	7
*RhoA*	CCCAGAAGTCTACGTCCCAAC	TCCTGTCCTGCTGTATCCCAC	102	1.99	19.91	0.54	2.71	9
*MOB*	CCTAAATGAGTGGATCGCTGTG	TGGTATTCATACTTCGGTCCTGC	132	1.94	21.85	0.49	2.24	3
*RPS15*	GTGGAAGTCAAGCCGGAGAT	AATCTTGACGAGTGAGTGGCG	110	1.82	16.46	0.48	2.92	10
*RPL17*	CGGAGTTGGTCGTTGTGCC	CCTTGACATCGGCGTTGCT	123	1.83	16.49	0.39	2.36	5
*UBQ2*	CAAGATTCCCGAGACCTATCC	CAGCCCATTGGTCCTTGAGTAT	125	2.23	21.03	0.53	2.52	6
*TubA*	CGAGCAGTCTGTATGTTGAGC	TTCCTCCATTCCCTCACCG	129	2.11	19.37	0.62	3.20	11
*TubB*	TCTCCGAGCAGTTTACCGCTATG	GTACTCGCTCACCAAGTCGTTC	125	1.98	16.74	0.83	4.96	14
*GAPDH*	CAAGCCAGCAACTTACGACGAG	GTGTCGCCGTTGAAATCAGTC	123	2.00	19.28	0.84	4.36	13
*BAG*	TCGTGGAATCGAACCTGTCCTTG	CATGAGCTGTAGGTGCTTGGTG	116	1.98	–	–	–	–

**FIGURE 4:**
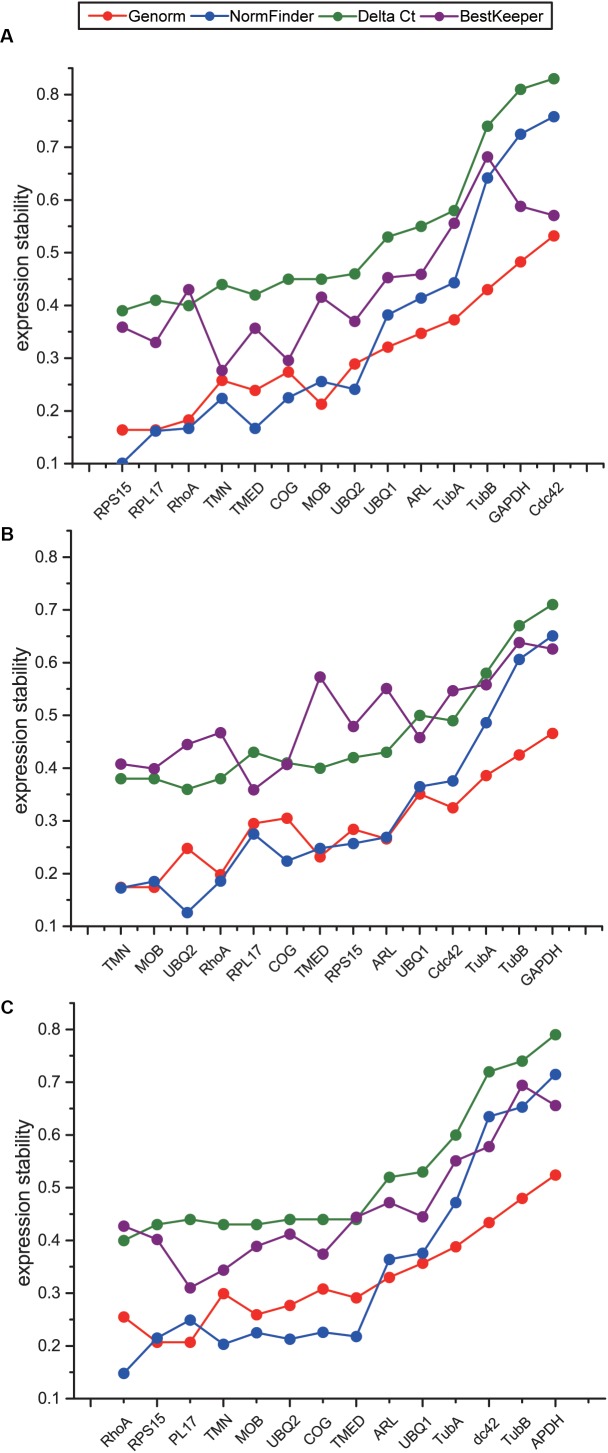
Comparison of expression stability of 14 candidate reference genes using Genorm, NormFinker, Delta Ct, and BestKeeper under temperature stress **(A)**, salinity stress **(B)**, and the combination of temperature and salinity stresses **(C)**. The sequence of genes on *X* axis indicates the comprehensive results obtained with RefFinder, according to the order from the most stable genes to the least stable genes.

To comprehensively evaluate the expression stability of the candidate reference genes, we used online software RefFinder (Xie et al., 2011), which is integrated with four programs including geNorm, NormFinder, Delta Ct, and BestKeeper, to screen stable reference genes suitable for temperature stress, salinity stress, and combined stresses. Although the ranks of the 14 candidate reference genes obtained by four programs were not exactly the same, the overall trends were similar. Four genes including *GAPDH, CDC42, TubA* and *TubB* were repeatedly identified as the least stable genes both in four different programs and all three datasets (Figure 4A–C), indicating that these four genes should not be selected as reference genes for temperature and salinity challenges. Our results showed that the most stable reference genes under temperature, salinity and combined stresses were not the same. *RPS15, RPL17* and *RhoA* were suggested as the top three stable reference genes under temperature stress, *TMN, MOB* and *UBQ2* under salinity stress, and *RhoA, RPS15* and *RPL17* under the combined stress (Figure 4A–C). As the most stable reference genes selected, *TMN* and *RhoA* were among the novel candidate reference genes from RNA-Seq results.

### Target Gene Expression Analysis

**FIGURE 5:**
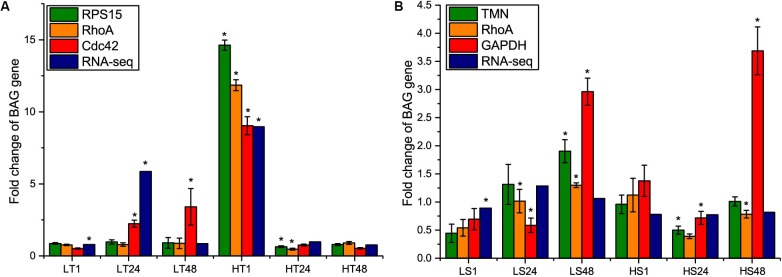
Comparison of relative expression levels of *BAG* gene normalized by the most stable reference gene (*RPS15* and *TMN*) and the least stable gene (*CDC42* and *GAPDH*) selected in temperature and salinity stress dataset separately, and the most stable reference gene (*RhoA*) selected in the combined dataset, with fold change values obtained from RNA-Seq results. **(A)** temperature stress, **(B)** salinity stress. LT, low temperature; HT, high temperature; LS, low salinity; HS, high salinity. The asterisks (^∗^) show the statistical difference (*p* < 0.05) between a treatment group and its corresponding control group.

To further re-evaluate the validity of selected reference genes, the relative expression level of *BAG* gene under temperature and salinity stresses was normalized with the most and least stable genes in temperature or salinity stress datasets, and the most stable gene in combined dataset. Subsequently, we compared the normalization results with the fold change value obtained from RNA-Seq results. After the temperature stress, the expression level of *BAG* showed exactly the same results regarding the direction and statistical significance of gene expression when normalized by *RPS15* and *RhoA*, consistently showing significant changes at 24 and 48 h of high temperature exposure (Figure 5A). When *CDC42* was used as the reference gene, the expression level of *BAG* was overestimated at 24 and 48 h after low temperature stress, and did not show significant change at 24 h under high temperature stress (Figure 5A). *BAG* expression profiles obtained from RNA-Seq were similar with that from RT-qPCR normalized by *RPS15* and *RhoA* at 48 h after low temperature stress and 1, 48 h after high temperature stress (Figure 5A).

After salinity stress, we found that the changing amplitude of *BAG* expression normalized by the least stable gene *GAPDH* was higher than that of the other normalizers at 48 h of high salinity stress (Figure 5B). The consistency of other three standardization methods was much lower than the results of temperature stress. Using *TMN* as reference gene, *BAG* expression was significantly up-regulated at 48 h in low salinity stress and down-regulated at 24 h in high salinity stress. Using *RhoA* as the reference gene, *BAG* expression was significantly up-regulated at 24, 48 h in low salinity stress and down-regulated at 48 h in high salinity stress. RNA-Seq results revealed that *BAG* gene was only differentially expressed after 1 h of low salinity stress (Figure 5B).

## Discussion

Gene expression analysis based on RNA-Seq is increasingly providing novel findings in the underlying molecular and cellular mechanisms for environmental adaptation and/or accommodation in invasive species (Lockwood and Somero, 2011; Jones et al., 2015). In addition to find DEGs to reveal the involved biological processes and metabolic pathways during *C. savignyi*’s response to temperature and salinity stresses, our results obtained in this study also confirmed that RNA-Seq was an effective method and provided new resources for the identification of stably expressed reference genes used for RT-qPCR. Although the previous study had selected stably expressed reference genes for stress response in *C. savignyi* (Huang et al., 2016), that study was just based on 10 commonly used reference genes referring to other related studies, and the recommended genes from other studies may not include the most stable references genes suitable for our present research. Our genome-wide analysis successfully identified novel reference genes, such as *RhoA* as the most stable one for the whole dataset and two novel reference genes *TMN* and *MOB* as the top two stable genes for salinity stress.

Although reference genes were generally selected from housekeeping genes and the definition of them were usually vague and even mixed, there were still minor differences between them. Two factors, expression universality among different tissues and expression stability under different conditions, should be considered simultaneously for housekeeping genes, so one important role of them was to define the minimal set of genes needed for organism’s survival (Koonin, 2000). The reference genes refer in particular to internal controls used in qRT-PCR, and expression stability was the prerequisite for reference genes. In the present study, completely different reference genes were recommended for temperature and salinity stresses, suggesting that gene stability varied greatly under different abiotic stresses. Thus, reference gene selection should be considered for each specific experimental design. Several studies found that housekeeping genes were compact with short length compared with tissue-specific genes driven by selection for economy during evolution (Vinogradov, 2004; Eisenberg and Levanon, 2013). Our study did not find the correlation between expression stability and gene length; however, we observed that the genes with a highest expression level under temperature and salinity stresses had shortest length. A universal relationship between expression level and gene compactness was also revealed in *Homo sapiens, Caenorhabditis elegans, Drosophila melanogaster*, and *Arabidopsis thaliana* (Carmel and Koonin, 2009). These highly expressed genes, either through environmentally induced or owing to background expression, might be caused by natural selection for minimum energy consumption and time expenditure in the mRNA transcription process to maintain normal functional metabolisms after environmental changes.

In addition to expression stability, the gene expression abundance should also be taken into consideration when selecting reference genes from RNA-Seq data, and genes with a low expression level were not considered as candidate reference genes because of low amplification efficiency and accuracy. Zhao et al. (2017) suggested that an FPKM value of 50–1000 should be adopted as the cut-off threshold for reference gene selection from RNA-Seq data. However, in our present RT-qPCR experiment, the average FPKM value of 14 candidate reference genes ranged from 15.94 (*COG*) to 1419.67 (*TubB*) (Table 1), and all these genes reached the normal range of mean Cq value from 16.46 (*RPS15*) to 23.12 (*COG*) (Table 2). Our results indicate that the range of FPKM value could also be relaxed under the premise that an appropriate concentration of the cDNA template was used in RT-qPCR.

Candidate reference genes are generally associated with or involved in basic biological processes, cellular components or molecular functions. For example, the selected stably expressed genes in different developmental stages of grapevine (*Vitis vinifera* L.) were involved in biological processes such as synthesis, degradation, folding, catabolism of proteins (González-Agüero et al., 2013), the significantly enriched pathways or GO terms of candidate reference genes in pregnant women were macromolecular complex, organelle, actin cytoskeleton (Chim et al., 2017), and the candidate reference genes in human non-melanoma skin cancers were involved in maintaining critical cellular activities including the citrate cycle, proteasome, RNA polymerase, protein export and ribosome activities (Hoang et al., 2017). The selected reference genes in our study are mainly associated with ribosome system, GTP binding, transcription and translation (Figure 1B). Ribosome is the important subcellular organelle where protein synthesis process occurs in all cellular organisms, and the expression of structural proteins constituting ribosome is highly stable. In a meta-analysis of 13,629 human gene array samples, 13 of the top 15 genes identified as the most stable reference genes were related to ribosomal structural proteins (de Jonge et al., 2007). The stability of ribosomal protein genes was confirmed in this study through RT-qPCR, and *RPS15* and *RPL17*, which code for the small and large subunit of ribosome respectively, were among the most stable reference genes identified under temperature stress and the combined stress (Figure 4A,C).

In addition to show constant expression level, the transcript abundance of ideal reference genes should be similar with those of target genes. *RPS15* and *RPL17* showed the highest expression level among the tested genes with the mean Cq values of 16.46 and 16.49 (Table 2), indicating that these two genes were suitable for normalizing the high expression target genes. For the moderate expression target genes, *RhoA* also with a moderate expression level and highly stable expression profile was a good alternative normalizer. *RhoA* was also identified as suitable reference gene in neuroendocrine tumors of the lung (Niu et al., 2016), and in porcine skeletal muscle at 26 different developmental stages (Walter et al., 2016). However, we have not found that the other two novel reference genes *TMN* and *MOB* are used as internal controls in gene expression analysis.

To clarify the impact of selected reference genes on relative expression analysis, we analyzed the transcript level of *BAG* gene, which is conserved and ubiquitously expressed in higher eukaryotes (Bitzer et al., 2016). *BAG*, as a chaperone protein, can interplay with heat shock 70 protein (*Hsp70*) and play important roles in organism’s response to heat stress (Corduan et al., 2009; Echevarría-Zomeño et al., 2016). If the standard of both fold change > 2 and *p*-value < 0.05 were adopted in the RNA-Seq analysis, *BAG* gene was up-regulated at 24 h after low temperature stress and at 1 h after high temperature, and not differentially expressed after salinity stress (Figure 5). Using RT-qPCR, we found that the use of different reference genes significantly altered the results of *BAG* expression, indicating the importance of selection of stable reference genes to assure the accuracy of gene expression results.

It is well known that RNA-Seq data can be seriously influenced by many factors, such as sample preparation and sequencing platform (Zhao et al., 2017). Thus, the batch effect has become a great challenge for identifying DEGs among different but related RNA-Seq experiments to avoid unnecessary repetitions and waste of time and money. It would be possible to carry out an integrative or comparison analysis among experiments/batches if the same genes were stably expressed in different databases. Therefore, in addition that the stably expressed reference genes can be used to normalize the target gene expression level for RT-qPCR, they could also be used as aligners to integrate different RNA-Seq datasets to possibly eliminate the batch effect. This strategy proposed here could largely solve the problems associated with batch/experimental variation when using RNA-Seq to study large-scale gene expression.

## Conclusion

We used the whole transcriptome dataset based on RNA-Seq to screen stably expressed reference genes for the model invasive ascidian *C. savignyi* under temperature and salinity stresses. A set of eight novel candidate reference genes with low variance of expression, as well as six commonly used reference genes, were subjected to a systematic analysis of expression stability using RT-qPCR by several methods. We found that the expression levels of 14 reference genes based on RNA-Seq was significantly correlated with that of RT-qPCR. Based on multiple analyses, we recommended the use of *RPS15* and *RPL17* and novel reference gene *RhoA* for gene expression analysis under temperature stress, and *TMN, MOB*, and *UBQ2* for salinity stress studies in *C. savignyi*. Our results clearly showed that the commonly used reference genes such as *TubA, TubB* and *GAPDH* should not be used as internal controls for gene expression analysis. When we used reference genes with differed stability, as expected we obtained contrasting results and conclusions, thus suggesting the importance of selection of proper reference genes and associated normalization methods. The validated reference genes in this study are expected to serve as reliable resources to further investigate molecular mechanisms of evolutionary adaptation and/or accommodation at the population level using the fast and accurate RT-qPCR method.

## Data Accessibility

The RNA-Seq raw sequence data was deposited in the National Center for Biotechnology Information (NCBI) Sequence Read Archive (SRA) database under the accession numbers SRP152910.

## Author Contributions

AZ and XH conceived the study. XH and SL designed the experiments. XH conducted the experiments, analyzed the data, and wrote the manuscript. All authors contributed to the revisions of this manuscript.

## Conflict of Interest Statement

The authors declare that the research was conducted in the absence of any commercial or financial relationships that could be construed as a potential conflict of interest.

## Footnotes

1 http://www.premierbiosoft.com/primerdesign/index.html
