# Supplementary material for: Genome-Wide Identification and Evaluation of New Reference Genes for Gene Expression Analysis Under Temperature and Salinity Stresses in Ciona savignyi
Source: Front Genet. 2019 Feb 12;10:71. doi: 10.3389/fgene.2019.00071 (PMC6380166; doi:10.3389/fgene.2019.00071)
Supplement: Supplementary file 1 [file Table_1.DOCX]

**Table S1** Statistics of the transcriptome sequencing data and mapping rate of 45 ascidian samples

| Sample | Clean Reads | Clean Bases | Q30 | Mapped reads % | Mapped  reads |
| --- | --- | --- | --- | --- | --- |
| LT1_2_1 | 16308711 | 2.45G | 97.23% | 80.00 | 13046969 |
| LT1_2_2 | 16308711 | 2.45G | 92.51% |  |  |
| LT1_5_1 | 14496408 | 2.17G | 97.34% | 82.92 | 12020422 |
| LT1_5_2 | 14496408 | 2.17G | 92.95% |  |  |
| LT1_6_1 | 15271238 | 2.29G | 97.01% | 84.87 | 12960700 |
| LT 1_6_2 | 15271238 | 2.29G | 91.42% |  |  |
| LT 24_1_1 | 20722397 | 3.11G | 97.25% | 83.50 | 17303201 |
| LT 24_1_2 | 20722397 | 3.11G | 92.26% |  |  |
| LT 24_3_1 | 29732331 | 4.46G | 97.38% | 87.24 | 25938486 |
| LT 24_3_2 | 29732331 | 4.46G | 92.43% |  |  |
| LT 24_4_1 | 14210739 | 2.13G | 97.23% | 81.40 | 11567542 |
| LT 24_4_2 | 14210739 | 2.13G | 92.29% |  |  |
| LT 48_1_1 | 16320002 | 2.45G | 97.32% | 87.45 | 14271842 |
| LT 48_1_2 | 16320002 | 2.45G | 94.22% |  |  |
| LT 48_4_1 | 15467411 | 2.32G | 96.95% | 81.56 | 12615220 |
| LT 48_4_2 | 15467411 | 2.32G | 93.21% |  |  |
| LT 48_5_1 | 21869256 | 3.28G | 97.23% | 84.93 | 18573559 |
| LT 48_5_2 | 21869256 | 3.28G | 92.46% |  |  |
| C1_1_1 | 29639837 | 4.45G | 97.34% | 83.28 | 24684056 |
| C1_1_2 | 29639837 | 4.45G | 92.83% |  |  |
| C1_5_1 | 26246159 | 3.94G | 97.30% | 78.13 | 20506124 |
| C1_5_2 | 26246159 | 3.94G | 91.59% |  |  |
| C1_6_1 | 24967701 | 3.75G | 97.31% | 84.04 | 20982856 |
| C1_6_2 | 24967701 | 3.75G | 92.17% |  |  |
| C24_3_1 | 15400167 | 2.31G | 97.33% | 79.31 | 12213872 |
| C24_3_2 | 15400167 | 2.31G | 92.05% |  |  |
| C24_2_1 | 14041101 | 2.11G | 97.00% | 79.06 | 11100894 |
| C24_2_2 | 14041101 | 2.11G | 91.51% |  |  |
| C24_6_1 | 13488512 | 2.02G | 97.07% | 77.95 | 10514295 |
| C24_6_2 | 13488512 | 2.02G | 93.64% |  |  |
| C48_2_1 | 16510552 | 2.48G | 96.95% | 76.22 | 12584343 |
| C48_2_2 | 16510552 | 2.48G | 93.41% |  |  |
| C48_3_1 | 16956818 | 2.54G | 97.00% | 79.35 | 13455235 |
| C48_3_2 | 16956818 | 2.54G | 92.43% |  |  |
| C48_6_1 | 13666062 | 2.05G | 97.23% | 77.10 | 10536534 |
| C48_6_2 | 13666062 | 2.05G | 92.87% |  |  |
| HT1_4_1 | 20712500 | 3.11G | 97.23% | 83.40 | 17274225 |
| HT 1_4_2 | 20712500 | 3.11G | 91.99% |  |  |
| HT 1_5_1 | 20354854 | 3.05G | 97.34% | 85.28 | 17358619 |
| HT 1_5_2 | 20354854 | 3.05G | 92.11% |  |  |
| HT 1_6_1 | 16781506 | 2.52G | 97.47% | 83.84 | 14069615 |
| HT 1_6_2 | 16781506 | 2.52G | 92.68% |  |  |
| HT 24_1_1 | 18357704 | 2.75G | 97.56% | 84.02 | 15424143 |
| HT 24_1_2 | 18357704 | 2.75G | 92.87% |  |  |
| HT 24_2_1 | 16168705 | 2.43G | 97.50% | 79.10 | 12789446 |
| HT 24_2_2 | 16168705 | 2.43G | 94.47% |  |  |
| HT 24_3_1 | 17398509 | 2.61G | 97.60% | 81.49 | 14178045 |
| HT 24_3_2 | 17398509 | 2.61G | 94.45% |  |  |
| HT 48_1_1 | 14439905 | 2.17G | 97.13% | 79.85 | 11530264 |
| HT 48_1_2 | 14439905 | 2.17G | 92.66% |  |  |
| HT 48_2_1 | 21012942 | 3.15G | 97.50% | 84.01 | 17652973 |
| HT 48_2_2 | 21012942 | 3.15G | 93.24% |  |  |
| HT 48_3_1 | 16542613 | 2.48G | 97.49% | 82.84 | 13703901 |
| HT 48_3_2 | 16542613 | 2.48G | 92.32% |  |  |
| LS1_1_1 | 17235148 | 2.59G | 97.47% | 85.75 | 14779139 |
| LS1_1_2 | 17235148 | 2.59G | 92.47% |  |  |
| LS1_2_1 | 16452520 | 2.47G | 97.55% | 87.15 | 14338371 |
| LS1_2_2 | 16452520 | 2.47G | 92.70% |  |  |
| LS1_3_1 | 21394828 | 3.21G | 97.50% | 84.95 | 18174906 |
| LS1_3_2 | 21394828 | 3.21G | 92.62% |  |  |
| LS24_1_1 | 24782086 | 3.72G | 96.60% | 86.64 | 21471199 |
| LS24_1_2 | 24782086 | 3.72G | 90.77% |  |  |
| LS24_2_1 | 17387702 | 2.61G | 96.72% | 81.48 | 14167500 |
| LS24_2_2 | 17387702 | 2.61G | 91.55% |  |  |
| LS24_3_1 | 18272455 | 2.74G | 96.64% | 84.60 | 15458497 |
| LS24_3_2 | 18272455 | 2.74G | 93.27% |  |  |
| LS48_1_1 | 15889445 | 2.38G | 97.09% | 75.14 | 11939329 |
| LS48_1_2 | 15889445 | 2.38G | 93.91% |  |  |
| LS48_2_1 | 14954438 | 2.24G | 97.48% | 80.81 | 12084681 |
| LS48_2_2 | 14954438 | 2.24G | 94.20% |  |  |
| LS48_3_1 | 13468866 | 2.02G | 97.13% | 80.27 | 10811459 |
| LS48_3_2 | 13468866 | 2.02G | 93.11% |  |  |
| HS1_1_1 | 18027499 | 2.7G | 97.51% | 86.87 | 15660488 |
| HS 1_1_2 | 18027499 | 2.7G | 92.82% |  |  |
| HS 1_2_1 | 20977533 | 3.15G | 97.54% | 83.41 | 17497360 |
| HS 1_2_2 | 20977533 | 3.15G | 92.32% |  |  |
| HS 1_3_1 | 21125170 | 3.17G | 97.55% | 83.69 | 17679655 |
| HS 1_3_2 | 21125170 | 3.17G | 92.89% |  |  |
| HS 24_1_1 | 36466329 | 5.47G | 97.53% | 82.31 | 30015435 |
| HS 24_1_2 | 36466329 | 5.47G | 92.62% |  |  |
| HS 24_2_1 | 29934030 | 4.49G | 97.59% | 81.56 | 24414195 |
| HS 24_2_2 | 29934030 | 4.49G | 93.13% |  |  |
| HS 24_3_1 | 20467975 | 3.07G | 97.55% | 82.87 | 16961811 |
| HS 24_3_2 | 20467975 | 3.07G | 93.86% |  |  |
| HS 48_4_1 | 16733398 | 2.51G | 97.41% | 80.23 | 13425205 |
| HS 48_4_2 | 16733398 | 2.51G | 93.77% |  |  |
| HS 48_5_1 | 19317112 | 2.9G | 96.57% | 81.34 | 15712539 |
| HS 48_5_2 | 19317112 | 2.9G | 92.09% |  |  |
| HS 48_6_1 | 17488675 | 2.62G | 96.56% | 82.92 | 14501609 |
| HS 48_6_2 | 17488675 | 2.62G | 91.80% |  |  |
